# Resistance Economy and New Population Policy in Iran

**Published:** 2017-01-28

**Authors:** Jalal Poorolajal

**Affiliations:** ^1^ Department of Epidemiology and Noncommunicable Diseases Research Center, School of Public Health, Hamadan University of Medical Sciences, Hamadan, Iran.

In recent years, population policies have been the focus of special attention among politicians and policymakers. Iran's population has been growing constantly during the past half century ^1^; so that the population has relatively doubled from 1975 to 1995 and has jumped over the last two decades. However, the fertility rate has decreased dramatically from 6.24 in 1975 to 3.95 in 1995 and 1.72 in 2016 (Figure 1). Accordingly, the fertility rate in Iran has fallen to less than 2.0 during the last decade. The fertility rate below replacement level is a serious demographic problem. To overcome this problem, the general policies of the population were issued in May 2013, including 14 clauses. According to the first clause, the fertility rate must increase above the replacement level^2^. However, it is not determined how much the fertility rate should be. Lack of attention to this critical issue may again result in uncontrollable and unpredictable population growth.

The fertility rate for high-income countries is usually taken as roughly approximate to the level of replacement fertility (2.1 in the UK, for example)^3^. In this case, an average of two children will replace all mothers and fathers, if the same number of boys and girls are born and all girls survive to the end of reproductive age. However, mortality, the unbalanced sex ratio at birth, and those women who remain childless should be taken into account. Therefore, the replacement fertility rate should be actually higher than 2.0 to stabilize population and compensate this depletion. That means a substantial proportion of women should have three or more children. The replacement fertility ranges from 2.5 to 3.3 in low- and middle-income countries depending on the situation^4^. In Iran, the under-five mortality rate is about 18 per 1000 live births and maternal mortality rate is about 24.6 per 100,000 live births^5^. In addition, the primary infertility is estimated between 2.8% and 3.4%^6^. Consequently, an average replacement fertility around 2.1 to 2.2 will provide a sustainable population for Iran.

**Figure 1:**
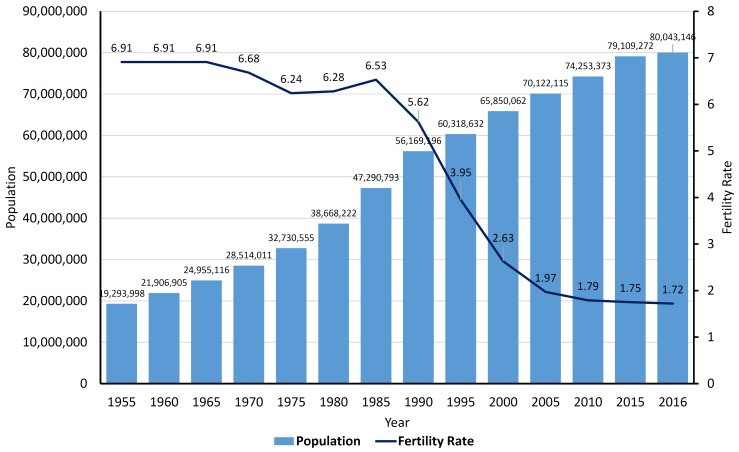
Trend of population growth and fertility rate in Iran from 1955 to 2016

In February 2013, the general policies of "resistance economy" were issued by the supreme leader of Iran, including 24 clauses^7^. The main objective of these policies is to develop a pattern of domestic economic on the basis of social values and norms, national resources and highly qualified workforce in order to reduce the vulnerability of the country against international sanction and even turn these pressures into opportunities. This is a paradoxical and sophisticated issue. On the one hand, Iranian population is aging due to low fertility rates, therefore, we need to increase the fertility rate above the replacement fertility level. On the other hand, we need to improve the economic status, inversely associated with fertility rate. In other words, gross domestic product increases as total fertility rate decreases and vice versa^8^ (Figure 2).

**Figure 2:**
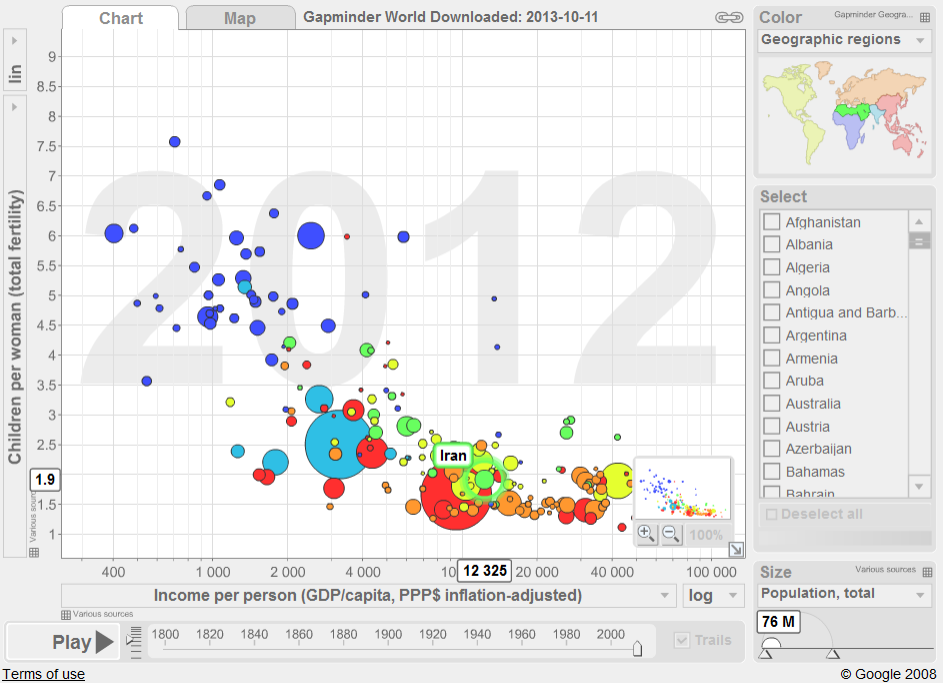
The correlation between economic growth and total fertility rate for all countries from 1800 to 2012, using Gapminder software

There is no doubt that fertility rate should increase above the replacement level, but we have to define a stringent population policy and determine an accurate level of replacement fertility to reach a sustainable population with reasonable growth rate. In such situation, we may expect to have a progressive economic and provide a "resistance economy" against international sanction; otherwise, we will back to the past two decades with unpredictable and unplanned population growth that may result in substantial and inevitable damage to the economic growth.

## Acknowledgements

The author declares that there is no conflict of interest.
